# A novel framework to estimate cognitive impairment via finger interaction with digital devices

**DOI:** 10.1093/braincomms/fcac194

**Published:** 2022-07-28

**Authors:** Ashley A Holmes, Shikha Tripathi, Emily Katz, Ijah Mondesire-Crump, Rahul Mahajan, Aaron Ritter, Teresa Arroyo-Gallego, Luca Giancardo

**Affiliations:** nQ Medical, Cambridge, MA 02142, USA; Center for Precision Health, School of Biomedical Informatics, University of Texas Health Science Center at Houston, Houston, TX 77030, USA; nQ Medical, Cambridge, MA 02142, USA; nQ Medical, Cambridge, MA 02142, USA; nQ Medical, Cambridge, MA 02142, USA; Division of Neurocritical Care, Department of Neurology, Brigham & Women’s Hospital, Boston, MA 02115, USA; Cleveland Clinic Lou Ruvo Center for Brain Health, Cleveland Clinic, Las Vegas, NV 89106, USA; nQ Medical, Cambridge, MA 02142, USA; Center for Precision Health, School of Biomedical Informatics, University of Texas Health Science Center at Houston, Houston, TX 77030, USA

**Keywords:** keystroke dynamics, digital biomarkers, cognition, clinical subdomains, machine learning

## Abstract

Measuring cognitive function is essential for characterizing brain health and tracking cognitive decline in Alzheimer’s Disease and other neurodegenerative conditions. Current tools to accurately evaluate cognitive impairment typically rely on a battery of questionnaires administered during clinical visits which is essential for the acquisition of repeated measurements in longitudinal studies. Previous studies have shown that the remote data collection of passively monitored daily interaction with personal digital devices can measure motor signs in the early stages of synucleinopathies, as well as facilitate longitudinal patient assessment in the real-world scenario with high patient compliance. This was achieved by the automatic discovery of patterns in the time series of keystroke dynamics, i.e. the time required to press and release keys, by machine learning algorithms. In this work, our hypothesis is that the typing patterns generated from user-device interaction may reflect relevant features of the effects of cognitive impairment caused by neurodegeneration. We use machine learning algorithms to estimate cognitive performance through the analysis of keystroke dynamic patterns that were extracted from mechanical and touchscreen keyboard use in a dataset of cognitively normal (*n* = 39, 51% male) and cognitively impaired subjects (*n* = 38, 60% male). These algorithms are trained and evaluated using a novel framework that integrates items from multiple neuropsychological and clinical scales into cognitive subdomains to generate a more holistic representation of multifaceted clinical signs. In our results, we see that these models based on typing input achieve moderate correlations with verbal memory, non-verbal memory and executive function subdomains [Spearman’s *ρ* between 0.54 (*P* < 0.001) and 0.42 (*P* < 0.001)] and a weak correlation with language/verbal skills [Spearman’s *ρ* 0.30 (*P* < 0.05)]. In addition, we observe a moderate correlation between our typing-based approach and the Total Montreal Cognitive Assessment score [Spearman’s *ρ* 0.48 (*P* < 0.001)]. Finally, we show that these machine learning models can perform better by using our subdomain framework that integrates the information from multiple neuropsychological scales as opposed to using the individual items that make up these scales. Our results support our hypothesis that typing patterns are able to reflect the effects of neurodegeneration in mild cognitive impairment and Alzheimer’s disease and that this new subdomain framework both helps the development of machine learning models and improves their interpretability.

## Teaser

Holmes and Tripathi *et al.* report moderate to good correlations of typing-based machine learning models to estimate cognitive performance with the clinical subdomains of verbal memory, non-verbal memory and executive function subdomains and a fair correlation with language/verbal skills.

## Graphical Abstract

**Graphical abstract:**
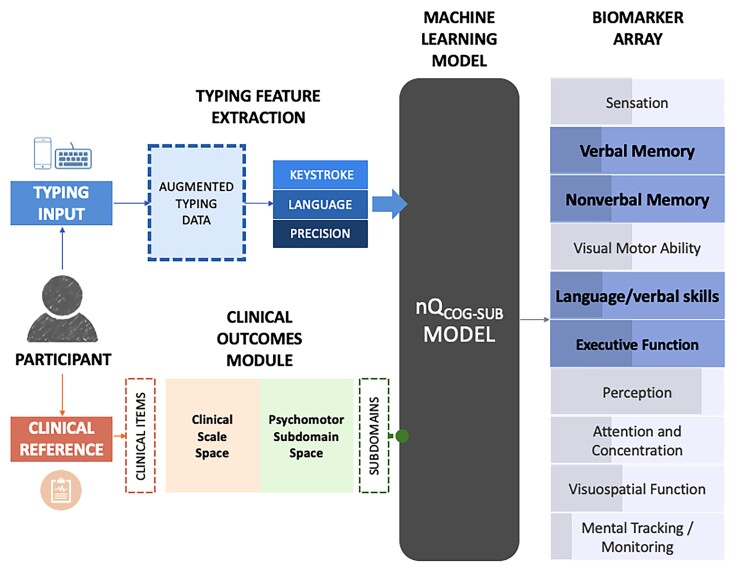

## Introduction

The complex and multifaceted nature of cognitive functioning poses real challenges when evaluating cognitive decline in a clinical context.^1^ Currently, the main approach used to characterize and quantify an individuals’ cognitive function involves a combination of clinical examination and psychometric tools.^2^ These tools mainly comprised questionnaires and clinical scales that include a variety of items in the form of patient-reported outcomes, focused clinical observations and small standardized tasks targeting specific skills and processes related to cognition.^3^ In general, the interpretation of these tests emphasizes the overall scale score with limited consideration of cognitive subdomains, which may lead to overly simplified clinical conclusions.^4^ Moreover, the limited variety of test options and the overlap between them increases the time required to complete an assessment and the likelihood that the testing does not provide a holistic view of cognitive state at the subdomain level.

Cognitive and neuropsychiatric assessments are typically administered during on-site clinical visits. While these clinical tools provide some level of standardization to the clinical evaluation, their administration and interpretation are subjective and dependent on provider experience and patient cooperation.^5^ In addition to their subjective and episodic nature, current standards for cognitive impairment screening and evaluation may have difficulty providing the level of granularity required to detect and quantify mild manifestations of disease and changes in a patients’ cognitive state.^6–8^ Digital technologies have introduced an opportunity to tackle some of these limitations.^9^

The widespread use of personal electronics has positioned typing among the most frequent activities of our daily living. The current reliance on technology allows the possibility of leveraging data from a users’ natural interaction with their devices to generate useful clinical insights in an unobtrusive manner.^9^ Passive monitoring maximizes a patients’ compliance^10^ as natural interactions with electronic devices can provide quasi-continuous information. The current work aims to demonstrate that changes in an individual’s keystroke patterns can be detected to indicate psychomotor and cognitive impairments.

Typing relies on the coordination and integration of multiple psychomotor processes such as cognition sensory feedback, as well as gross and fine motor control.^11^ Neurodegeneration in Alzheimer’s disease, even in mild cognitive impairment (MCI) or earlier stages directly affects psychomotor processing and impacts patients’ typing performance.^12,13^ Our previous work in Parkinson’s disease focused on the characterization of fine motor impairment through the analysis of content agnostic information derived from natural keystroke patterns.^14–16^ Other groups have also used this data source to train models to find associations with Parkinson’s disease rating scales,^14–18^ or detection of cognitive impairment.^19,20^ However, to our knowledge, no published work has developed models for cognitive impairment associated with specific cognitive subdomains or with graded level of severity. Because typing not only relies on fine motor control but also engages several cognitive faculties, such as memory and language, in this work, we show that passively collected keystroke patterns can be used not only to generate scores that approximate to cognitive status but that they can also identify impairments in specific cognitive subdomains.

Although digital biomarkers offer promise of more objective, quantitative and continuous ways to monitor symptoms, these solutions rely upon the processing of unique digital datasets using complex technologies that challenge traditional frameworks of clinical assessment and evidence generation within the healthcare ecosystem. In fact, interpreting the output of digital technologies has become a main barrier to their adoption for clinical care and clinical trials, in particular for those that rely on machine learning-based systems. Cognitive biomarkers should reflect the multifaceted nature of the phenomenon under study, allowing physicians and researchers to interpret its outcome at the subdomain level.

**Figure 1:**
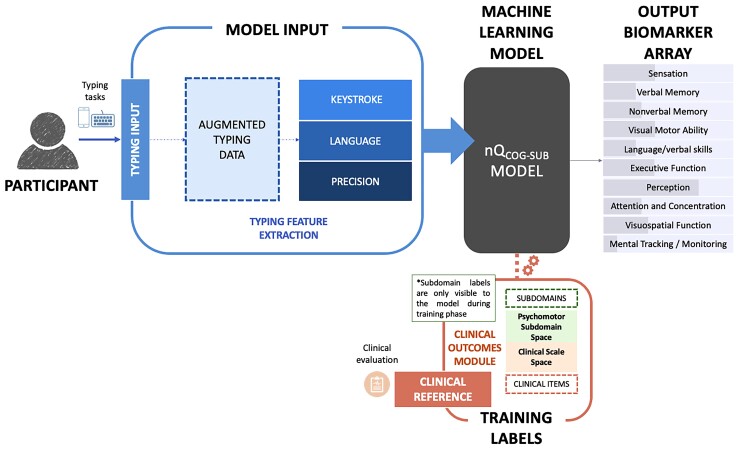
**Schema of experimental framework.** The proposed methodology uses machine learning algorithms to match keystroke patterns collected from participants interaction with mechanical and touchscreen keyboards to their cognitive state defined by standard neuropsychological assessments. The model is designed to ingest a feature vector derived from the raw data captured during semi-controlled typing tasks. During the training phase, the nQi_COG−SUB_ model uses a subdomain-level representation of the cognitive state of the patient as reference to connect the typing inputs to the level of impairment observed on each cognitive subdomain. For a given typing input, the model outputs a numeric estimate of the level of impairment for each of the cognitive subdomains under study. Information from clinical assessment or subdomain is only visible to the models during the training phase.

In this work, our main aim is to (i) introduce a framework to integrate multiple neuropsychological and clinical scales to generate cognitive subdomain representation of multifaceted clinical assessments. Our secondary aims are to (ii) demonstrate how machine learning models trained on passively collected keystroke patterns collected from touchscreen devices and mechanical keyboards generate scores significantly associated with cognitive impairment and (iii) demonstrate how the proposed cognitive subdomain framework leads to stronger associations between model outputs and cognitive subdomains than an alternative approach to model training based on individual neuropsychological and cognitive scale items (Fig. 1).

## Materials and methods

### Study population

This study was conducted as a natural history observational study assessing keyboard performance in 120 well-characterized participants currently enrolled in the Center for Neurodegeneration and Translational Neuroscience (CNTN), a collaborative enterprise between the Cleveland Clinic Lou Ruvo Center for Brain Health (CCLRCBH) and the University of Nevada Las Vegas.^21^ The CNTN enrols and characterizes this cohort of 120 individuals with early stage Alzheimer’s disease, Parkinson’s disease, and a cognitively normal control group. A subset (*N* = 77) of these individuals, with complete clinical assessments and typing tasks data, were included in the experiments presented in this article. Individuals undergo annual neuropsychological testing, structural and molecular imaging, and clinical examination, including a typing assessment completed during the clinical visit. After each annual assessment the participant is assigned a diagnosis based on consensus criteria. For this portion of the study we included all individuals who conducted typing assessments and were diagnosed with early stage cognitive impairment (MCI or dementia) or were considered cognitively normal, regardless of the presence or absence of Parkinson’s disease. For the purposes of modelling, participants were grouped into two main age- and gender-matched categories: 38 cognitively impaired (comprised participants with MCI, MCI/Alzheimer’s disease, Alzheimer’s disease, Parkinson’s disease-MCI) and 39 cognitively normal (comprised Parkinson’s disease participants without cognitive impairment and healthy controls) (see Table 1). Initial diagnostic classifications were achieved using the National Institute on Aging and Alzheimer’s Association criteria; confirmation of diagnosis was achieved via a consensus conference of physicians and neuropsychologists. Amyloid PET scan status was known but did not influence the diagnosis. Cognitively impaired and normal participants showed no statistically significant differences in age and years of education according to the Kruskal–Wallis test. Similarly, no statistically significant differences in sex were found according to χ^2^ test.

**Table 1:** Summary of clinical and demographic data

	Cognitively impaired	Cognitively normal	
Subjects #	38	39	
Age, mean (std)	73.6 (6.4)	71.1 (7.3)	*P* = 0.08^a^
Males #	23	20	*P* = 0.56^b^
Years of education, mean (std)	16.8 (2.7)	16.4 (2.1)	*P* = 0.33^a^
MoCA, mean (std)	23.0 (3.9)	27.4 (2.0)	*P* < 0.001^a^

^a^ Kruskal–Wallis test.

^b^ χ^2^ test.

### Clinical outcomes module

**Figure 2:**
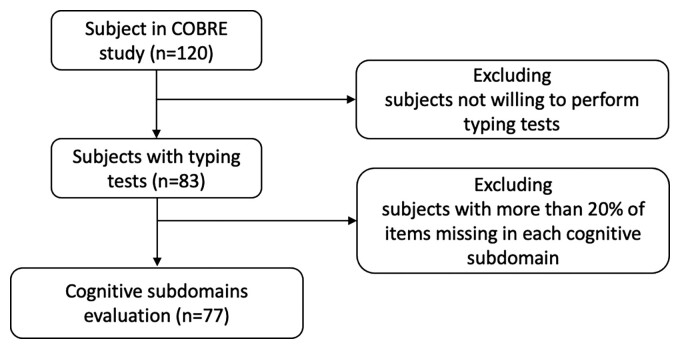
**Patient recruitment flowchart.** A total of 77 subjects were included in this study. Table 1 shows the summary of clinical and demographic data.

As shown in Table 2, we compiled nine different cognitive subdomains based on the literature. Each clinical item in the cognitive assessment was weighted and mapped to one (or more) of these nine cognitive subdomains (Fig. 2). The cognitive assessments included in our subdomain mapping and analysis are detailed in Table 2.^22–25^ All items from the MoCA were included and were accessed in the following grouped format as established by the CCLRCBH Center of Biomedical Research Excellence (COBRE)’s clinical data management group: (i) visuospatial/executive score (sum of Trail Making Test B task, clock-drawing task, 3D cube task); (ii) Naming score (3-item confrontation naming task); (iii) Attention score (sum of the Sustained Attention task, Serial Subtraction task, Digits Forward task, Digits Backward task); (iv) Language score (sum of the Phonemic Fluency task, Repetition of 2 Syntactically Complex Sentences task); (v) Abstraction score (2-item verbal abstraction task); (vi) Memory score (short-term memory recall task); and (vii) Orientation score (sum of Spatial orientation task, Temporal orientation task). Supplementary Table A1 contains a description of each item in each scale used, the original scoring system for that item, and the subdomain(s) to which each item was mapped. Note that the subdomain composition has been chosen a priori, uniquely based on existing literature, before attempting to train any type of predictive model. Information from clinical assessment or subdomain were used as training reference to the predictive models.

**Table 2:** Definition of cognitive subdomains and contributing scales

Subdomain	Scale items	Definition
Verbal memory	DRS2—Memory FAB—Lexical Fluency MoCA—Attention MoCA—Memory	The memory of words and/or other items regarding language.
Non-verbal memory	DRS2—Memory FAB—Lexical Fluency MoCA—Attention MoCA—Memory MoCA—Orientation	The memory of abstractions, pictures, concepts, directions, songs, etc. Does not include the memory of words/language.
Visual motor ability	FAB—Motor Series FAB—Conflicting Instructions FAB—Go-No-Go	Visuo-constructive function, the ability to copy and draw objects.
Language/verbal skills	FAB—Lexical Fluency MoCA—Naming MoCA—Language	Include receptive and productive abilities and the ability to understand language, access semantic memory, to identify objects with a name, and to respond to verbal instructions with behavioural acts.
Executive function	ADLQ—Self-Care DSR2—Initiation/Perseveration DRS2—Construction DRS2—Conceptualization FAB—Similarities FAB—Motor Series FAB—Conflicting Instructions FAB—Go-No-Go FAB—Prehension Behaviour MoCA—Visuospatial/Executive MoCA—Language MoCA—Abstraction	The set of processes that manifest control over other component cognitive abilities, such that cognitive resources can be effectively utilized to solve problems efficiently and plan for the future (reasoning and problem solving).
Perception	DRS2—Conceptualization FAB—Prehension Behaviour	Sensory info is processed and integrated. It can be assessed in terms of ability to recognize objects, sounds, and also for the intactness of the perceptual fields.
Attention and concentration	DSR2—Attention FAB—Conflicting Instructions FAB—Go-No-Go MoCA—Attention	Includes selective/sustained attention and divided attention, all of which have executive functioning components. Concentration falls under sustained attention.
Visuospatial function	MoCA—Visuospatial/Executive MoCA—Orientation	Involves identification of a stimulus and its location.
Mental tracking/monitoring	MoCA—Attention	Involves being able to recite the alphabet, months backwards, and letter-number alternation.

**Figure 3:**
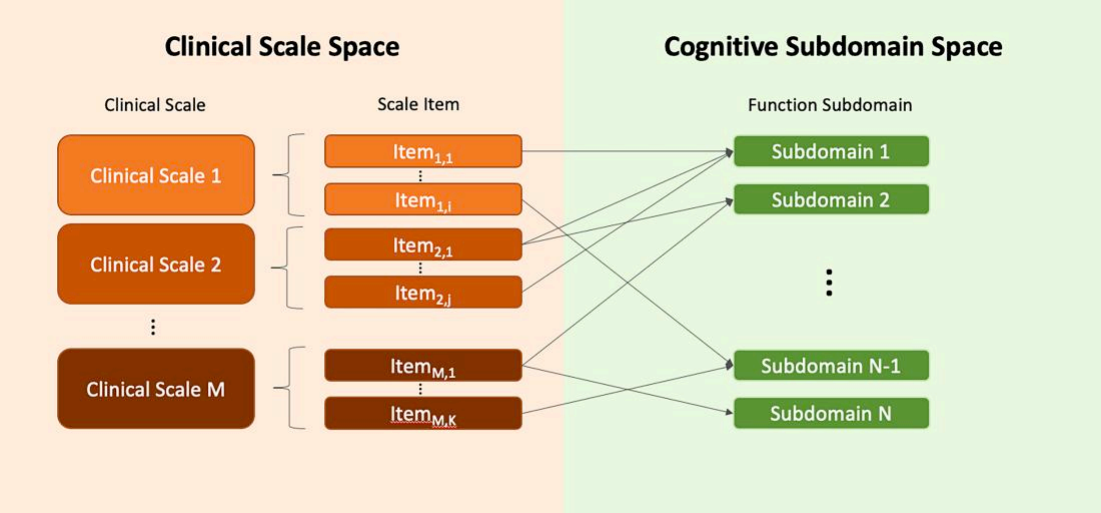
**Clinical Outcomes Module.** This framework transforms the results from the standard neuropsychological assessments measured in the clinical scale space into a simplified representation of the multiscale information in the cognitive subdomain space. Based on their definition, clinical scale items are mapped to the corresponding subdomains of cognition that they measure, and their scores are normalized to generate a standardized and aggregated representation of the cognitive state of the individual.

To compare cognitive subdomains, we converted clinical items from each scale into a standardized range of [0, 1], where 0 represents no impairment and 1 represents the highest level of impairment (Fig. 3). Comparing clinical items on the same scale allows for a single directionality and a single severity range, both of which facilitate a direct comparison of cognitive domain severity. For some scales, the highest score for an item represents the highest level of impairment, whereas for other scales, it is the lowest score for an item which represents higher impairment. All scale items are converted such that a higher score represents more impairment, and therefore the subdomain scores also reflect this directionality. Formally:

A clinical scale *X* is made up of multiple items *x*_*i*_ such that *X* = *x*_1_ +  …  + *x*_*N*_. Each clinical scale item *x*_*i*_ is transformed into norm(*x*_*i*_) by dividing the item by the maximum possible value of that item:

(1) $$\mathrm{norm}(x_{i})=\frac{x_{i}}{(x_{i})\;}\mathrm{where}\;\mathrm{norm}(x_{i})\in [0,1]$$

A subdomain score *S* is calculated by first summing all normalized clinical scale items norm(*x*_*i*_) from all clinical scales for which the literature suggests the item measures the subdomain of impairment and then dividing by the number of valid items *M* mapped to that subdomain:

(2) $$\mathrm{norm}(S)=\frac{\sum x_{i}}{M}\;\mathrm{where}\;\mathrm{norm}(S)\in [0,1]\;$$

where *N* = The number of items in a clinical scale, *x_i_* = One item in a clinical scale taking on an integer value in [0, (*x_i_*)], (*x_i_*) = The maximum value of a clinical scale item, norm(*x_i_*) = A normalized clinical scale item taking on a value in [0, 1], *M* = The number of valid items (from any number of clinical scales) that map to a single subdomain, norm(*S*) = A normalized subdomain score taking on a value in [0, 1].

### Typing feature extraction

All subjects completed two tests in two paradigms: mechanical keyboard typing and smartphone touch keyboard typing. The mechanical keyboard typing tests were completed on a standardized laptop (Lenovo 330-15IGM running Windows 10) and consisted of a copying task and a description task. During the mechanical copying task (Task_mec_copy_), subjects were asked to transcribe the *Rainbow* passage,^26^ a standard phonetically balanced excerpt used in speech and language evaluations, using a standard word processor or texting application. The subjects were given 5 min and instructed to type as they normally do at home and left free to correct typing mistakes only if they wanted to. During the description task (Task_mec_des_), subjects were given 5 min to provide a free-typing description of the ‘Cookie Theft’ picture from the Boston Diagnostic Aphasia Examination^27^ on the mechanical keyboard (QWERTY Standard built in Lenovo 330-15IGM).

The smartphone typing tests were completed on a standardized mobile phone (LG K8 2018 running Android 7.1.2, using a touchscreen keyboard) and consisted of a copying task (Task_tch_copy_) and a simulated text conversation (Task_tch_conv_). During the touchscreen copying task, participants were asked to transcribe the *Grandfather* passage,^28^ a standard phonetically balanced excerpt used in speech and language evaluations, in a dedicated touchscreen text view within the nQ Medical mobile phone application. During the simulated text conversation, the subjects answered a series of three questions in a dedicated touchscreen text view within the nQ Medical mobile phone application. The questions were designed to gather information about participants’ state and familiarity with smartphone use to evaluate potential correlations between their keystroke patterns, their present mood and self-reported skill level.

During each typing task, the nQ Medical data collection software captures augmented typing data, an input comprised of multiple dimensions of finger-keyboard interactions including:

- Keystroke data, defined as timing of press and release events in a typing stream. $\left(P_{k_{n}},R_{k_{n}}\right)$
- Key location data, defined as the keyboard zone corresponding to each keystroke event $\left(Z_{k_{n}}\right)$
- Tap precision data, defined as the relative distance of the tap centre to the target key centre (only applies to touchscreen data) $\left(E_{k_{n}}=[E_{k_{nx}},E_{k_{ny}}]\right)$
- Key type data, defined as the key-content category corresponding to each keystroke event (alphanumeric, space, enter, punctuation, modifier, emoji) $\left(T_{k_{n}}\right)$
- Assisted typing events, defined as a log of autocorrect and usage of word suggestions provided by the keyboard review tool and predictive engine (only applies to smart keyboard data) (*A*_*e*_, *W*_*e*_)
- Typing session context, that may include details like session start time, the application hosting the typing session, characteristics of the device used to generate the typing session, metrics that monitor device state, etc. (*C*)

Augmented typing session data are assembled in nested variable-length arrays to generate raw keystroke tensors (*S*_*I*_):

(3) $$S_{I}=\{P_{k_{n}}^{I},R_{k_{n}}^{I},Z_{k_{n}}^{I},E_{k_{n}}^{I},T_{k_{n}}^{I},A_{e}^{I},W_{e}^{I},C^{I}\}$$

where *I* represents the session or, in this case, the typing task identifier, *k*_*n*_ refers to each unique keystroke within a typing stream, and *e* identifies smart keyboard events within a given session.

Different dimensions captured by raw keystroke tensors are combined to generate a series of primitive signals in the shape of enriched keystroke tensors. Enriched keystroke tensors are the result of successive transformations of the raw typing data structures. These transformations apply combinations of one or multiple data types to generate a series of primitive feature families that can be included in one of the following categories:

- Keystroke: Content agnostic analysis of the timing information of combinations of pressing and releasing keystroke events during a typing session.
- Language: Content agnostic analysis of text structure and complexity based on the length and distribution of words and the use of punctuation.
- Precision: This category gathers information about the use of backspace, the level of intervention of the autocorrect function in smart keyboards, as well as finger precision for each keystroke when tapping on a touchscreen keyboard.

Primitive signals belonging to each of these feature families are then reduced to a predefined size feature vector that will be used as input to the model.

### nQi_COG_ modelling

We used two type of machine learning models to generate scores able to predict cognitive status uniquely from the typing features described above generated from the tasks involving mechanical keyboards (Task_mec_copy,_ Task_mec_des_) and touch screens (Task_tch_copy_, Task_tch_conv_). The first approach, nQi_COG−SUB_*Jointly Optimized* model, attempts to learn all cognitive subdomains or clinical items at the same time by minimizing a joint loss, and it is based on extremely randomized trees (i.e. extra-trees).^29^ The second approach, nQi_COG−SUB_*Independently Optimized* model, attempts to learn all cognitive subdomains or clinical items independently and it is based on a Gradient Boosting Decision with tree gradient-based one-side sampling (GOSS) as implemented in the LightGBM package v. 3.1.1.^30^ While a plethora of other machine learning approaches exists, we selected these two as they have been shown to ac hieve excellent performance with problems involving feature engineering, like ours, as indicated by the number of citations (currently over 4000 per paper), and they allow to compare the predictive performance change when cognitive subdomains are used in lieu of clinical items.

The nQi_COG−SUB_*Jointly Optimized* model is a way of solving a ‘multi-output problem’ that leverages the correlation between outcomes (i.e. cognitive subdomains or clinical items) to improve predictive performance. The main drawback of this approach is that outcomes that are not predictable can drive down the performance of the model as a whole. The extra-trees model used in this work constructs an ensemble of decision trees. It applies the idea of randomness to split the nodes to reduce the variance. Any split made is evaluated by calculating a mean squared error function. We utilize the class ‘ExtraTreeRegressor’ from the scikit-learn library v. 0.24.2 and build an ensemble of 100 trees using a mean squared error loss function. We compensate for any missing feature by imputing the mean as the library does not directly support missing values.

In the nQi_COG−SUB_*Independently Optimized* model we solve the ‘multi-output problem’ by learning multiple targets independently, which requires a ‘cold-start’ for each of the outcomes, but avoiding well predictable outcomes to be negatively affected by less predictable ones. In addition, this approach allows us to include all of the subjects in the data set, as we do not have to discard subjects with missing clinical test data. We use the LightGBM package v. 3.1.1 for tree GOSS with 100 estimators and mean squared error loss function as in the previous model. In this case, all missing values are automatically handled by the gradient boosting approach.

No feature scaling was performed as both methods are based on decision trees, which are not sensitive to change in variance in the data. To avoid any chance of overfitting, all models where trained and tested with 10 repetitions of a 3-fold cross-validation strategy. At each iteration, the order of the samples was randomized to allow for identifying different folds and no data sample coming from the same subject appeared in the training and testing fold at the same time. The default optimization and other hyperparameters provided by the LightGBM (v. 3.1.1)^30^ and Scikit-learn (v. 0.24.2).^31^ While this might not lead to the highest performing model, it would avoid any chance of overfitting induced by manually tuning hyperparameters without using a validation split.^32^ We used a supervised approach for model development, i.e. the clinical subdomains labels were visible to the model only during the training phase.

In addition to the two nQi_COG−SUB_ models designed to tackle the ‘multi-output problem’, we also built the nQi_COG_ model following a ‘single-output problem’ design. The purpose of this model is to evaluate the performance of the multi-output approach versus the traditional single outcome design. This model is trained against the MoCA total score following the exact same model architecture and train-test strategy as the nQi_COG−SUB_*Independently Optimized*, i.e. a tree GOSS trained and tested using the same 10 repetitions of a 3-fold cross-validation strategy described previously and the default setup in the LightGBM (v. 3.1.1) implementation.^30^

### Evaluation

All outputs of the models were evaluated using Pearson’s *r* and Spearman *ρ* to test both linear and monotonic relationships between the models’ predictions versus clinical items and the models’ predictions versus the cognitive subdomain developed. Apart from the correlations, coefficient of determination (R2) and *P*-value representing its significance are calculated to analyze the performance of the regression models. Mean squared error (mse) is also calculated to estimate the overall error in the model’s prediction.

As shown in Table 1, our data set does not seem to have clear confounders between the cognitive impaired and cognitive normal groups; however, we performed an additional confounder analysis on the scores generated by the trained models. For each score in each model, we estimated the measure of association to the clinical subdomain with a linear regression model. Then, the same model was adjusted for age or sex. The change between the two is indicative of a potential confounding effect of the variables investigated and was computed as follows:

(4) $$\mathrm{change}=\frac{\left|a_{0}-a_{\mathrm{adj}}\right|}{\left|a_{\mathrm{adj}}\right|}$$

where *a*_0_ is the unadjusted coefficient and *a*_adj_ is the adjusted one. Both coefficients have been computed using ordinary least square models.

### Data availability statement

Anonymized data, not published in the article, will be shared on reasonable request from a qualified investigator.

## Results

In Table 3, we show the correlation of our two models with the proposed subdomains. The nQi_COG–SUB_*Independently Optimized* model can predict the scores of four of the nine subdomains with weak to moderate correlation in both Pearson’s *r* and Spearman’s *ρ* and a weak but statistically significant correlation with R2.^33^ Using the nQi_COG−SUB_*Jointly Optimized* model, we find the same statistical significant correlation, although with slightly lower coefficient of correlation.

**Table 3:** Correlation between subdomains and the predicted scores for nQi_COG−SUB_ Independently Optimized and nQi_COG−SUB_ Jointly Optimized models

	nQi_COG−SUB_*Independently Optimized* Model (LightGBM)					nQi_COG−SUB_*Jointly Optimized* Model (Extra Trees)				
	Pearson’s *r* (significance)	Spearman’s *ρ* (significance)	R2	Mean Squared Error (mse)	*n*	Pearson’s *r* (significance)	Spearman’s *ρ* (significance)	R2	Mean Squared Error (mse)	*n*
Verbal memory	0.508 (***)	0.504 (***)	0.258 (***)	0.017	61	0.516 (***)	0.454 (***)	0.266 (***)	0.015	61
Non-verbal memory	0.458 (***)	0.545 (***)	0.210 (***)	0.017	63	0.451 (***)	0.446 (***)	0.203 (***)	0.013	61
Executive Function	0.469 (***)	0.424 (***)	0.220 (***)	0.003	61	0.336 (**)	0.301 (*)	0.113 (**)	0.003	61
Language/verbal skills	0.262 (*)	0.303 (*)	0.069 (*)	0.012	61	0.222 (n.s.)	0.205 (n.s.)	0.049 (n.s.)	0.012	61
Mental tracking/monitoring	0.001 (n.s.)	0.059 (n.s.)	0.000 (n.s.)	0.025	68	0.348 (**)	0.286 (*)	0.121 (**)	0.016	61
Visual motor ability	0.030 (n.s.)	0.050 (n.s.)	0.001 (n.s.)	0.023	70	0.046 (n.s.)	0.141 (n.s.)	0.002 (n.s.)	0.022	61
Perception	−0.272 (*)	−0.188 (n.s.)	0.074 (*)	0.001	70	0.065 (n.s.)	0.084 (n.s.)	0.004 (n.s.)	0.000	61
Attention and concentration	−0.181 (n.s.)	−0.168 (n.s.)	0.033 (n.s.)	0.010	61	0.128 (n.s.)	0.152 (n.s.)	0.016 (n.s.)	0.010	61
Visuospatial function	0.218 (n.s.)	0.204 (n.s.)	0.048 (n.s.)	0.019	68	0.111 (n.s.)	0.164 (n.s.)	0.121 (**)	0.013	61

For the nQi_COG−SUB_*Independently Optimized* model, R2, Spearman’s *ρ* and Pearson’s *r* provide the same results for statistical significance, with verbal memory, non-verbal memory, and executive function reporting *P* < 0.001, language/verbal skills reporting *P* < 0.05, and the remaining subdomains reporting no statistical significance.

**Figure 4:**
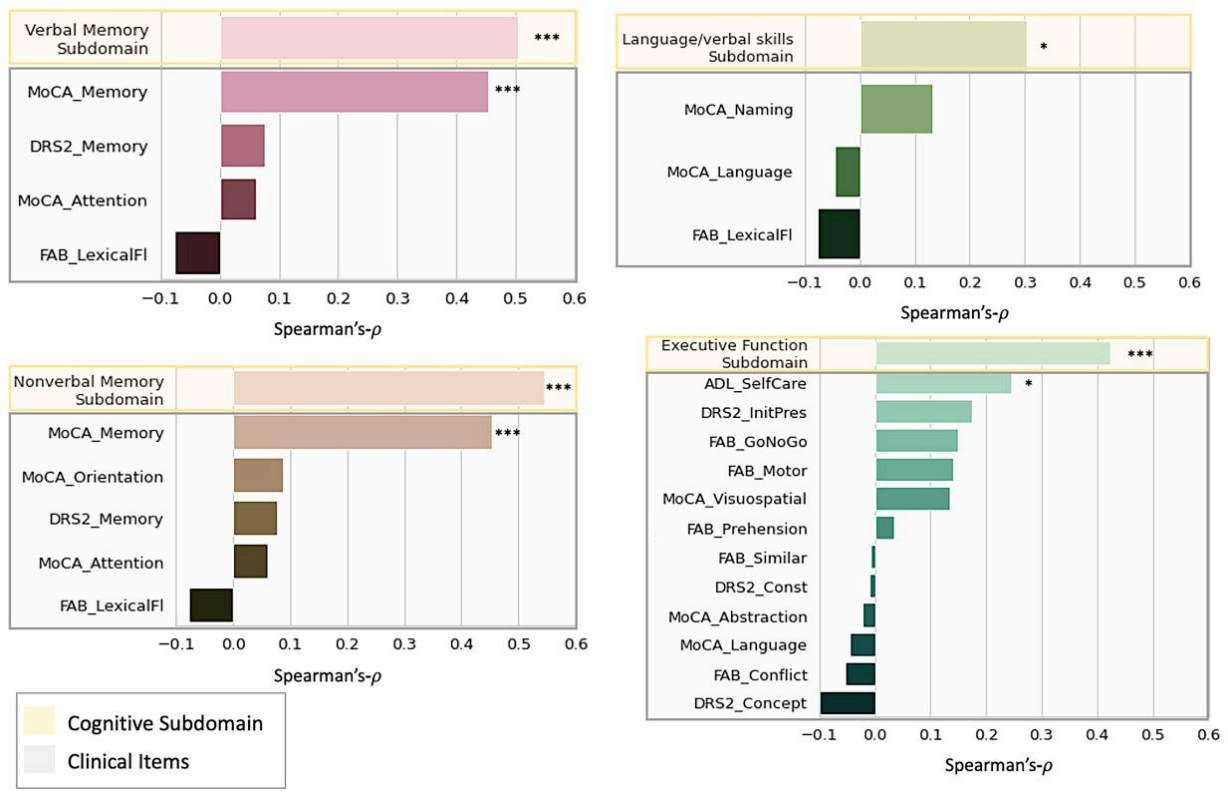
**Correlation between cognition and keystroke dynamic models.** In each panel, the nQi_COG−SUB_*Independently Optimized* model is trained and tested using a 10 repetitions of a randomized 3-fold cross-validation strategy on the cognitive subdomain (yellow background) and the scale components that make up the subdomain (grey background). We calculated the Spearman’s *ρ* between the model and each of the subdomains for a set of subjects. The number of subjects varied for subdomains ranging from 61 in the verbal memory, 63 in non-verbal memory, 61 in executive function and 61 in language/verbal skills. In all cases where the subdomains were composed of more than a single item, the model had higher correlations with subdomains compared with the individual items. Significance is noted as follows: *P* < 0.001 (***), *P* < 0.01 (**), *P* < 0.05 (*), and *P* ≥ 0.05 (). In this case, the *P*-value can be interpreted as the probability of an uncorrelated system producing datasets that have a correlation coefficient at least as extreme as the one observed in this data set. These findings were replicated also when using the *Jointly Optimized* model as shown in Supplementary Fig. A1. Note that the subdomain composition has been chosen a priori, before attempting to train any type of predictive model. Subdomain with Spearman’s *ρ* < 0.3 are not shown as the model did not have enough predictive ability to draw any conclusion. Full results are shown in Table 3

**Figure 5:**
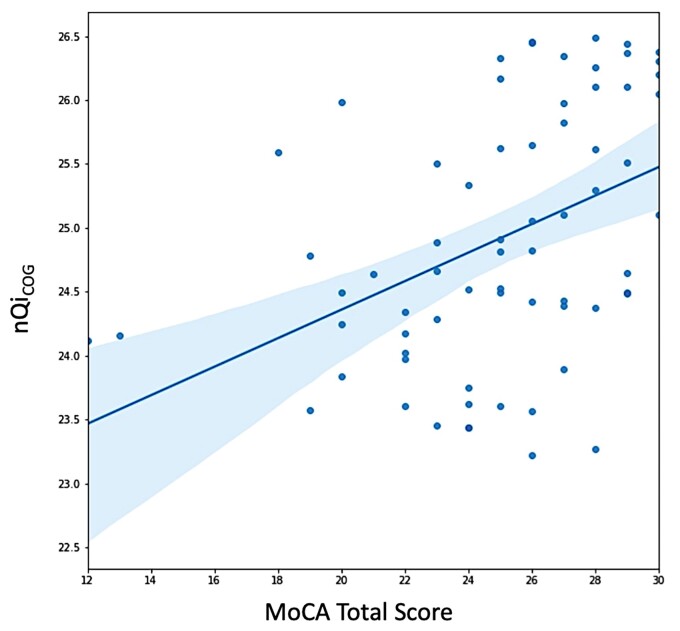
**Correlation between nQi_COG_ and MoCA.** The figure includes a scatter of the MoCA and nQi_COG_ sample pairs, as well as the line of best fit representing the relationship between the model output and the clinical reference. The shaded area represents the 95% confidence interval for the regressed line. Pearson’s *r* = 0.42***, Spearman’s *ρ* = 0.48*** and R2 = 0.18***. Significance is noted as follows: *P* < 0.001 (***), *P* < 0.01 (**) and *P* < 0.05 (*)

In Fig. 4, we compare performance of nQi_COG−SUB_*Independently Optimized* with a LightGBM-based architecture, when trained on the subdomains or on the individual clinical items that make up the subdomain. In all cases, using the subdomain as outcome for the model results in a better correlation than any of its constituent clinical items taken individually, in some cases very significantly, such as with executive function, where *ρ* = 0.42 for the subdomain but the best correlation in the individual clinical item space is *ρ* = 0.24. Looking at the results of the ‘single-output’ reference, the nQi_COG_, against the MoCA total score we observe a correlation of *ρ* = 0.48, which is slightly better than the best correlation achieved by the ‘multi-output’ models in the subdomain space (Fig. 5).

Evaluating sex and age as confounding factors for nQi_COG−SUB_*Independently Optimized* when trained for predicting verbal memory, non-verbal memory, executive function and language/verbal skills, i.e. the four subdomains that can be predicted with a weak to moderate correlation,^33^ we see no confounding effect in the majority of cases using a change cut-off of 10%^34^ in the corrected versus uncorrected model. The only exceptions are the language/verbal skills subdomain, where adjusting for age induces a change of 29%, and the non-verbal memory where adjusting for age induces a change of 13%. Confounder analysis results are shown in Supplementary Tables A2 and A3.

To generate more insights on what typing information is selected by the models to estimate the cognitive subdomains, we perform a Shapley Additive Explanations (SHAP) analysis^35^ using the independently optimized model (Supplementary Figs. A2 and A3). This allows us to estimate a relevance weight for each individual typing feature and each typing task. As individual typing features taken do not have an obvious interpretation, we have grouped them by type (i.e. precision, keystroke, language) and task. For each of the training iterations, we collect the SHAP values for the corresponding test folds. The collection process is repeated for each subdomain.

Overall, we observe there is a statistically significant connection between user-device typing patterns and their cognitive state. In addition to the correlation observed between the nQi_COG_ model and the total MoCA score, the subdomain-based approach suggests that there are specific facets of cognitive performance that seem to be more clearly reflected in participants’ typing patterns. Looking at the significance and strength of the correlation between the multi-output typing-derived biomarkers and each corresponding subdomain score, we see how verbal memory, non-verbal memory, executive function and language/verbal skills stood out, based on these results, as being more directly connected to the cognitive processes controlling how users type. Results appear to be independent of potential confounders, based on post-correction analysis. Feature importance analysis suggests that both mechanical and touchscreen typing inputs contribute similarly to the model predictions. The analysis based on feature families indicates language and keystroke features and more relevant than precision-based features in defining the model outputs.

## Discussion

We present a method that generates quantitative measures of cognitive status both at global and subdomain levels using the analysis of keystroke patterns extracted from computer and smartphone interactions. While other works have indicated that cognitive impairment impacts patients’ typing performance,^12,13^ this work is the first attempt to provide interpretable granular metrics directly extracted from the way our fingers interact with keyboards. Our findings open a pathway to the development of passive digital measurements that aim to provide more frequent, sensitive, and accessible ways to evaluate patients’ state than current clinical standards.

Today, cognitive evaluations often require patients to undergo a battery of neuropsychological assessments. Research suggests that clinical scales for cognitive screening, may be either too broad to detect specific subdomain impairment for certain conditions or too focused on disease specific aspects and thus they do not present a true picture of overall functional impairment.^36–40^ In addition, apart from being time-consuming for the patient and clinician, current neuropsychological testing results in a collection of assessments with a variety of independent and overlapping clinical items that are hard to interpret as a whole. One of the main contributions of this work is the introduction of the Clinical Outcomes Module, a tool that integrates the information from multiple standard assessments to generate an aggregate representation of the cognitive state that is presented at the subdomain level.

In the context of this work, this tool has allowed us to train our machine learning algorithms against a representation of the cognitive function deconstructed into functional subdomains. This way, we have been able to run parallel optimization of each of the typing-based algorithm outputs against different known aspects of cognitive decline. As different phenotypes of impaired cognition may manifest differently through typing, this approach based on multiple outputs is able to provide a more detailed representation of the impact of neurodegeneration expressed in users’ typing by enhancing the specific patterns that reflect functional impairment at the subdomain level.

From a biomarker understanding perspective, this approach has also allowed us to identify the aspects of cognition that, based on the results of this work, seem to be more relevant to typing. By looking at the correlations of each typing-based biomarker against their corresponding subdomain score, we observe that executive function, language/verbal skills, verbal memory and non-verbal memory are the components of cognitive performance that appeared to be better captured by daily typing patterns.^41^ These four cases achieved a statistically significant correlations ranging from weak to moderate, which indicates that these models have the potential to be used to evaluate cognitive status remotely on the patients’ digital devices. This could improve clinical research, clinical trials and routine care, as the cognitive status of the subject can be measured at a much higher frequency than what is normally carried out, at the subject’s home as opposed to the clinic and with minimal effort on the subject side, which can improve compliance compared with standard classic cognitive tests.

In all four cases, the typing-based outcome presented a stronger correlation against the clinical target when using the subdomain space versus the clinical scale item space for model optimization and evaluation. In addition to that, age and sex did not have a significant effect on the typing-based biomarkers. The only exceptions were sex for the language/verbal skills subdomain and non-verbal memory; however, these effects were small for non-verbal memory and affected language/verbal skills likely due to the fact that the model only achieved a weak correlation. The correlation observed in the reference model, the ‘single-output’ nQi_COG_, against the total MoCA score reveals a stronger relationship between the output and the total MoCA score than the correlation observed for the best performing jointly optimized model predictions and their corresponding subdomain scores. This balance in performance could be due to the natural correlation present between the overall MoCA score and the subdomain scores, as these are partially derived from MoCA components. The advantage of the subdomain decomposition is that it has the potential to reveal the aspects of cognition that seem to have a closer connection to typing. Still, the independently optimized model outperforms ‘single-output’ nQi_COG_ for verbal and non-verbal memory subdomains.

In this work, we compared and contrasted two tree-based machine learning models, one jointly optimized and another independently optimized. This analysis allowed us to evaluate if the correlation between the subdomains was strong enough to facilitate the learning phase of the jointly optimized model. However, this did not seem to be the case likely because some subdomains were not predictable from our feature set, which negatively impacted the theoretical advantages of the joint optimization as a whole.

The Clinical Outcomes Module introduced in this work has multiple potential applications. Here we present a use case for supervised optimization of typing-based biomarkers against different subdomains of cognition. However, this framework could be useful to support multiple areas in biomarker development other than algorithm building. For example, the Clinical Outcomes Module could be considered the first step towards the development of a tool to enhance clinical interpretability of cognitive testing as it provides an understanding of the weight or level of connection of different areas of cognition to a given biomarker. In addition, this tool could also be optimized to facilitate comparability and aggregation of different clinical data sets. Our view is that this approach would not be limited to cognitive characterization as it could be applied to other clinical domains such as behavioural or motor functions.

This study has some limitations. First, the features extracted require the use of a touchscreen device and a laptop, which might exclude some subjects. In addition, subjects with high degree of cognitive impairment are unlikely to be able to operate these devices. However, this group of patients is typically not the focus of clinical trials or monitoring by neurologists. For this analysis, we had access to a subset of the clinical scales available in the COBRE study and, in some instances, some of these samples were incomplete (i.e. missing scale items, missing data, etc.), which limited the information available to apply the scale to subdomain transformation. For future iterations of this work, we will explore the possibility of including additional clinical scales and items to enhance the robustness and accuracy of the subdomain score estimates. Finally, the relatively small size of our cohort does not allow the use of other machine learning techniques able to perform representation learning, such as deep neural networks. Additional data collection would also allow for independent testing in a separate cohort to further validate and test generalizability of the proposed methods, as well as discarding any biases in this limited data set leading to inflated prediction accuracy. These limitations will be addressed in future work.

We presented an approach that allows for the development of computational biomarkers that are directly comparable to known aspects of cognitive performance and therefore directly interpretable by expert neurologists. By relying on natural typing, this work leverages the frequency of users’ daily interactions with their personal devices to introduce an unobtrusive and quasi-continuous approach to characterize cognitive decline in the MCI-Alzheimer’s disease spectrum. Future works will aim to translate our learnings from the analysis of typing conducted within a semi-controlled environment to the real-world setting. Passive, quantitative, continuous, and objective tools can support precision medicine for cognitive characterization offering physicians and patients an accurate, frequent and less burdensome method of cognitive assessment and phenotyping.

## Supplementary Material

fcac194_Supplementary_Data

## Abbreviations

ADLQ =: Activities of Daily Living Questionnaire
CCLRCBH =: Cleveland Clinic Lou Ruvo Center for Brain Health
CNTN =: Center for Neurodegeneration and Translational Neuroscience
DRS2 =: Dementia Rating Scale-2
FAB =: Frontal Assessment Battery
GOSS =: gradient-based one-side sampling
MCI =: mild cognitive impairment
MoCA =: Montreal Cognitive Assessment
NIA-AA =: National Institute on Aging and Alzheimer’s Association
UNLV =: University of Nevada Las Vegas.
