# Supplementary material for: A novel framework to estimate cognitive impairment via finger interaction with digital devices
Source: Brain Commun. 2022 Jul 28;4(4):fcac194. doi: 10.1093/braincomms/fcac194 (PMC9356723; doi:10.1093/braincomms/fcac194)
Supplement: fcac194_Supplementary_Data [file fcac194_supplementary_data.pdf]

Supplementary Materials

A novel framework to estimate cognitive impairment via finger interaction with digital devices

Ashley A. Holmes[1,†], Shikha Tripathi[2,†], Emily Katz[1], Ijah Mondesire-Crump[1], Rahul Mahajan[1,3], Aaron Ritter[4], Teresa Arroyo-Gallego[1,‡] and Luca Giancardo[2,‡]

[†,‡] These authors contributed equally to this work.

Clinical Scale to Subdomain Mapping

Supplementary Table A1 Mapping between clinical scale items and cognitive subdomains

Item	Scoring System	Original Instrument	Item Description	Subdomains	Reference
Self-Care	0-100	Activities of Daily Living Questionnaire	The examiner is instructed to "score each item according to the subject's current level of ability relative to their customary performance prior to the onset of dementia symptoms." The items in the self-care subscale include eating, dressing, bathing, elimination, taking pills, and personal appearance.	Executive function	Johnson *et al.*[1]
Attention	37-0	Dementia Rating Scale - 2	This subscale consists of untimed tasks of digit span forward and backward, motor response to single commands, visual scanning ordered list reading, and visual figure matching.	Attention	Matteau *et al.*[2]
Initiation/ Perseveration	37-0	Dementia Rating Scale - 2	This subscale consists of tasks of time limited word generation to two different semantic categories.	Executive function	Matteau *et al.*[2]
Construction	6-0	Dementia Rating Scale - 2	This subscale involves the copying of five geometric figures of varying difficulty, and the writing of one's name.	Executive function	Matteau *et al.*[2]
Conceptualization	39-0	Dementia Rating Scale - 2	This subscale consists of tasks of identification of similarities between pairs of objects, recognition of similarities between objects (multiple-choice format), primed inductive reasoning, identification of nonmembers of semantic categories, identification of similarities and differences among simple geometric figures, and generation of a simple sentence using specified words.	Executive function, Perception	Matteau *et al.*[2]

Memory	25-0	Dementia Rating Scale - 2	This subscale consists of tasks of short-term recall of two sentences, recognition memory for words and geometric designs, and orientation for tor time (day, date, month, year), place (hospital, city), and current events (president, governor, mayor).	Nonverbal memory, verbal memory	Matteau *et al.*[2]
Similarities	3-0	Frontal Assessment Battery	The examiner names two/three words and subject must give an answer to the question "in what way are they alike?". The first two times contain two words, while the third and final time contains three words.	Executive function	Dubois *et al.*[3]
Lexical Fluency	3-0	Frontal Assessment Battery	The subject says as many words as they can beginning with the letter 'S' (any words except surnames or proper nouns) within 60 seconds.	Nonverbal memory, language, verbal memory	Dubois *et al.*[3]
Motor Series	3-0	Frontal Assessment Battery	The examiner, seated in front of the subject, performs alone three times using the left hand the series of Luria motions of "fist–edge-palm." Then the examiner prompts the subject: "Now, with your right hand do the same series, first with me, then alone." The examiner performs the series three times in total with the subject. Then the subject must perform the series three times alone while the examiner observes the subject's actions.	Executive function, visual motor ability	Dubois *et al.*[3]
Conflicting Instructions	3-0	Frontal Assessment Battery	The examiner tells the subject: "Tap twice when I tap once." The examiner does a series of three trial runs to be sure that the subject has understood the instructions (1-1-1). The examiner tells the subject "Tap once when I tap twice." The examiner does a series of three trial runs to be sure that the subject has understood the instructions (2-2-2). The examiner now performs the actual following series: 1-1-2-1-2-2-2-1-1-2.	Executive function, visual motor ability, attention	Dubois *et al.*[3]
Go-No-Go	3-0	Frontal Assessment Battery	The examiner tells the subject: "Tap once when I tap once." The examiner does a series of three trial runs to be sure that the subject has understood the instructions (1-1-1). The examiner tells the subject "Do not tap when I tap twice." The examiner does a series of three trial runs to be sure that the subject has understood the instructions (2-2-2). The examiner now performs the actual following series: 1-1-2-1-2-2-2-1-1-2.	Executive function, visual motor ability, attention	Dubois *et al.*[3]
Prehension Behavior	3-0	Frontal Assessment Battery	The examiner tells the subject: "Tap once when I tap once." The examiner does a series of three trial runs to be sure that the subject has understood the instructions (1-1-1). The examiner tells the subject "Do not tap when I tap twice." The examiner does a series of three trial runs to be sure that the subject has understood the instructions (2-2-2). The examiner now performs the actual following series: 1-1-2-1-2-2-2-1-1-2.	Executive function, perception	Dubois *et al.*[3]
Visuospatial/ Executive Score	5-0	Montreal Cognitive Assessment	Sum of the Trail Making Test B task (The subject is presented with 10 circles, 5 of which contain one letter A through E, and 5 of which contain the numbers 1 through 5. The subject, beginning at 1, must connect the circles with arrows, 1-A-2-B-3-...-5-E), Clock-Drawing task (The subject must draw a clock at ten past eleven, including the contour of the clock, the numbers, and the hands), and 3-D Cube task (the subject must copy a drawing of a 3-D cube).	Executive function, visuospatial ability	Kang *et al.*[4] Wang *et al.*[5]

Naming	3-0	Montreal Cognitive Assessment	The subject is presented with images of three animals and the subject must identify the animals by name.	Language	Kang *et al.*[4]<br>Wang *et al.*[5]
Attention	6-0	Montreal Cognitive Assessment	Sum of the Sustained Attention task (The examiner reads a list of 29 letters to the subject. The subject must tap with their hand at each letter A), Serial Subtraction task (Starting at 100, the subject must subtract 7 from 100 at least 4 times.), Digits Forward task (Given a series of 5 numbers, the subject must repeat the numbers in the forward order), and Digits Backward task (Given a series of 3 numbers, the subject has to repeat them in the backward order).	Nonverbal memory, attention, verbal memory, mental tracking	Kang *et al.*[4]<br>Wang *et al.*[5]
Language	3-0	Montreal Cognitive Assessment	Sum of the Phonemic fluency task (The subject must name as many words as possible that begin with the letter F in one minute) and Repetition of 2 Syntactically Complex Sentences task (The examiner reads two sentences to the subject, and the subject must repeat the sentences)	Executive function, language	Kang *et al.*[4]<br>Wang *et al.*[5]
Abstraction	2-0	Montreal Cognitive Assessment	The subject is given two words and asked to identify the similarity between the words, for two sets of words.	Executive function	Kang *et al.*[4]<br>Wang *et al.*[5]
Memory	5-0	Montreal Cognitive Assessment	The examiner reads a list of 5 words at a rate of one per second. The subject must recall 5 words read to them with no cue at the end of the test.	Nonverbal memory, verbal memory	Kang *et al.*[4]<br>Wang *et al.*[5]
Orientation	6-0	Montreal Cognitive Assessment	The subject must give the current date, month, year, day, place, and city.	Nonverbal memory, visuospatial ability	Kang *et al.*[4]<br>Wang *et al.*[5]

nQi$_{COG\text{-}SUB}$ *Jointly-Optimized* Results

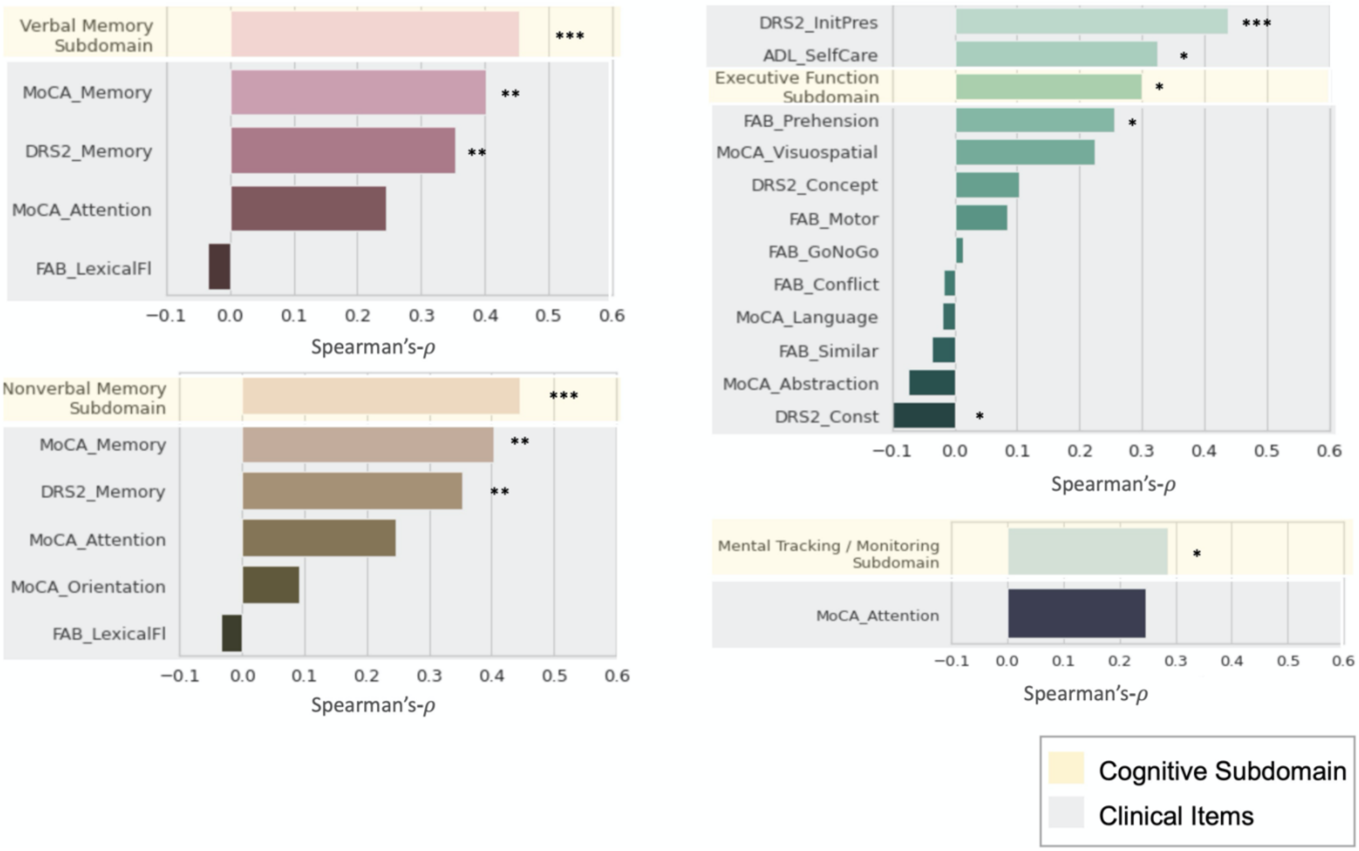

Supplementary Figure A1 Correlation between cognition and keystroke dynamic models. In each panel, the nQi$_{COG\text{-}SUB}$ *Jointly-Optimized* model is trained and tested using a leave-one-subject-out strategy on the cognitive subdomain (yellow background) and the single items that make up the subdomain (grey background). In all cases where the subdomains were composed of more than a single item, the model had higher correlations with subdomains compared to the individual items. The number of subjects remained the same for all subdomains (n=61). Significance is noted as follows: $p<0.001$(***), $p<0.01$(**), $p<0.05$(*), and $p\geq0.05$( ). In this case, the p-value can be interpreted as the probability of an uncorrelated system producing datasets that have a correlation coefficient at least as extreme as the one observed in this dataset. These findings were replicated also when using the *Independently-Optimized* model. Note that the subdomain composition have been chosen a priori, before attempting to train any type of predictive model. Subdomain with Spearman's *rho* < 0.3 are not shown as the model did not have enough predictive ability to draw any conclusion. Full results are shown in Table 3.

Confounder Analysis

We do not include age and sex as input features in our models. It is important to account for any confounding effects they might have on our model's predictions. Therefore, we analyze age and sex for their potential confounding effects. For every cognitive subdomain, we utilize the predictions from the two models. These predictions and the corresponding ground truth are used to construct a linear regression model. The model produces the coefficient representing a measure of association between the prediction and the ground truth values. The same predictions, when used with age or sex as one of the other inputs in the simple regression model, produce another set of coefficients. We compare the coefficients for the predictions from these models to observe any changes. Any changes equal to or greater than the value of 10%, are an indicator of a potential confounder. The values for the age variable are normalized before conducting the analysis.

$$Model_0 : a_{00} predictions \rightarrow subdomain$$

$$Model_{age} : a_{0,age} predictions + a_{1,age} age \rightarrow subdomain$$

$$Model_{sex} : a_{0,sex} predictions + a_{1,sex} sex \rightarrow subdomain$$

For any simple linear regression can be identified by the equation:

$$a_1 x_1 + a_2 x_2 + \cdots + constant \rightarrow y$$

where,

y: dependent variable; x_i: independent variable; a_i: measure of association

Following the equation of linear regression, we compare the change between the coefficients a_{00} and $a_{0,age}$ to determine if age can be a potential confounder. Similarly, when we compare a_{00} and $a_{0,gender}$ we determine if sex can be a potential confounder. The change is calculated with the formula below.

$$change_{age} = \frac{abs(a_{00} - a_{0,age})}{abs(a_{0,age})}$$

$$change_{sex} = \frac{abs(a_{00} - a_{0,sex})}{abs(a_{0,sex})}$$

Since, we are interested in the magnitude of change, we consider the absolute values of change.

Supplementary Table A2 Results of analyzing 'Age' as a potential confounder in the nQiCOG-SUB Independently-Optimized and nQiCOG-SUB Jointly-Optimized models

**Subdomain**	**% Change: nQi$_{COG-SUB}$ *Independently-Optimized***	**% Change: nQi$_{COG-SUB}$ *Jointly-Optimized***
Verbal Memory	5.590	5.689
Nonverbal Memory	12.931	4.816
Language/verbal skills	29.664	40.348
Executive Function	1.620	6.718

Supplementary Table A3 Results of analyzing 'Sex' as a potential confounder in the nQiCOG-SUB Independently-Optimized and nQiCOG-SUB Jointly-Optimized models

**Subdomain**	**% Change: nQi$_{COG-SUB}$ *Independently-Optimized***	**% Change: nQi$_{COG-SUB}$ *Jointly-Optimized***
Verbal Memory	1.258	1.150
Nonverbal Memory	4.598	1.682
Language/verbal skills	1.608	1.659
Executive Function	1.044	0.878

Feature Importance

To better understand the our models predictions we calculate feature importance for nQi$_{COG-SUB}$ *Independently-Optimized.* We use Shapley Additive Explinations (SHAP) analysis method and calculate the feature importances. For each of the training iteration we collect the SHAP values for the corresponding test sets. The collection process is repeated for each subdomain. These summed up feature importance values are then grouped by tasks Task, Task2, Task3, and Task4 as shown in Figure 2. Task1 and Task3 correspond to typing data collected from a touchscreen source in uncontrolled setting. Task2 and Task4 correspond to

typing data collected from mechanical source in a controlled setting. We also group the feature importances by the feature classes backspace, keystroke, and language as shown in Figure 3.

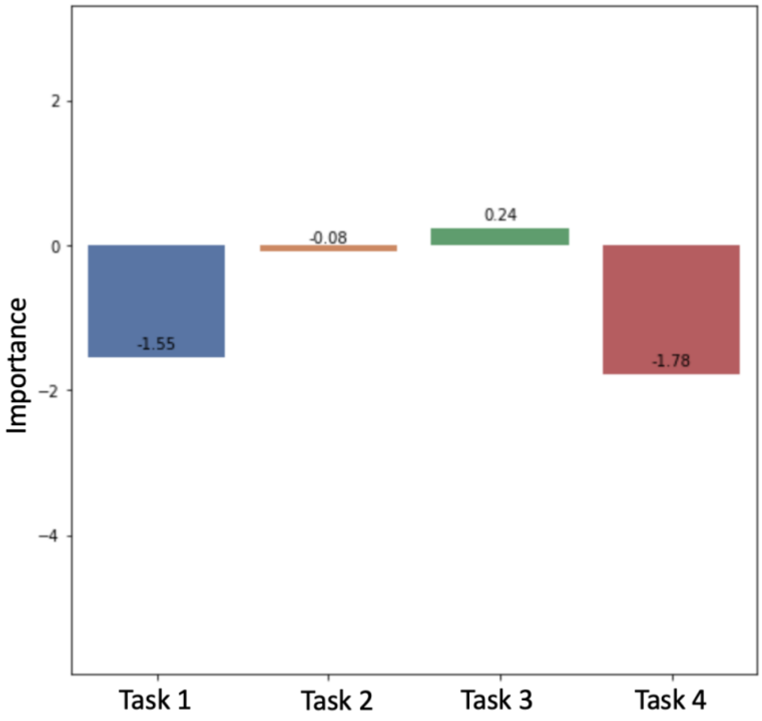

Supplementary Figure A2 Feature importances grouped by tasks.

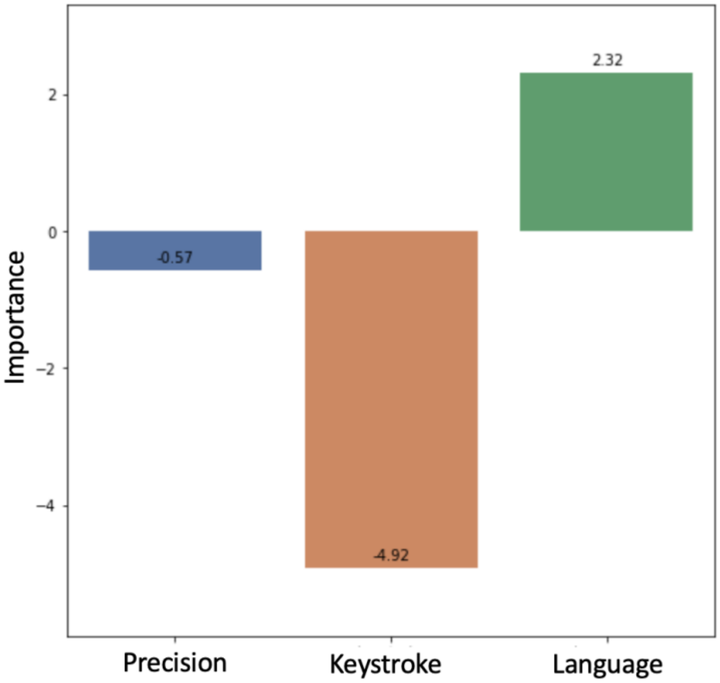

Supplementary Figure A3 Feature importances grouped by feature family.

References

1. Johnson N, Barion A, Rademaker A, Rehkemper G, Weintraub S. The Activities of Daily Living Questionnaire: A Validation Study in Patients with Dementia. *Alzheimer Disease & Associated Disorders*. 2004;18(4).

2. Matteau E, Dupré N, Langlois M, et al. Mattis Dementia Rating scale 2: Screening for MCI and dementia. *American Journal of Alzheimer's Disease and other Dementias*. 2011;26(5):389-398. doi:10.1177/1533317511412046

3. Dubois B, Slachevsky A, Litvan I, Pillon B. The FAB: A frontal assessment battery at bedside. *Neurology*. 2000;55(11):1621-1626. doi:10.1212/WNL.55.11.1621

4. Kang JM, Cho YS, Park S, et al. Montreal cognitive assessment reflects cognitive reserve. *BMC Geriatrics*. 2018;18(1):1-8. doi:10.1186/s12877-018-0951-8

5. Wang YX, Zhao J, Li DK, et al. Associations between cognitive impairment and motor dysfunction in Parkinson's disease. *Brain and Behavior*. 2017;7(6). doi:10.1002/brb3.719
